# Focus on Paediatric Restrictive Cardiomyopathy: Frequently Asked Questions

**DOI:** 10.3390/diagnostics13243666

**Published:** 2023-12-14

**Authors:** Mattia Zampieri, Chiara Di Filippo, Chiara Zocchi, Vera Fico, Cristina Golinelli, Gaia Spaziani, Giovanni Calabri, Elena Bennati, Francesca Girolami, Alberto Marchi, Silvia Passantino, Giulio Porcedda, Guglielmo Capponi, Alessia Gozzini, Iacopo Olivotto, Luca Ragni, Silvia Favilli

**Affiliations:** 1 Pediatric Cardiology, Meyer Children’s University Hospital IRCCS, 50134 Florence, Italysilvia.favilli@meyer.it (S.F.); 2 Cardiomyopathy Unit, Careggi University Hospital, 50134 Florence, Italy; 3 Local Health Unit, Outpatient Cardiology Clinic, 84131 Salerno, Italy; 4 Cardiovascular Department, San Donato Hospital, 52100 Arezzo, Italy; 5 Pediatric Cardiology and Adult Congenital Heart Disease Program, Department of Cardio—Thoracic and Vascular Medicine, IRCCS Azienda Ospedaliero—Universitaria di Bologna, 40138 Bologna, Italy

**Keywords:** restrictive cardiomyopathy, paediatric

## Abstract

Restrictive cardiomyopathy (RCM) is characterized by restrictive ventricular pathophysiology determined by increased myocardial stiffness. While suspicion of RCM is initially raised by clinical evaluation and supported by electrocardiographic and echocardiographic findings, invasive hemodynamic evaluation is often required for diagnosis and management of patients during follow-up. RCM is commonly associated with a poor prognosis and a high incidence of heart failure, and PH is reported in paediatric patients with RCM. Currently, only a few therapies are available for specific RCM aetiologies. Early referral to centres for advanced heart failure treatment is often necessary. The aim of this review is to address questions frequently asked when facing paediatric patients with RCM, including issues related to aetiologies, clinical presentation, diagnostic process and prognosis.

## 1. Introduction

The recently published guidelines defined cardiomyopathy as “a myocardial disorder in which the heart muscle is structurally and functionally abnormal, in the absence of coronary artery disease, hypertension, valvular disease, and congenital heart disease sufficient to cause the observed myocardial abnormalities” [1]. This definition applies to both children and adults and makes no a priori assumptions about aetiology (which can be familial/genetic or acquired) or myocardial pathology.

Currently, cardiomyopathies are distinguished into hypertrophic cardiomyopathy (HCM), dilated cardiomyopathy (DCM), arrhythmogenic cardiomyopathy (ACM), restrictive cardiomyopathy (RCM) and a novel entity that has been recently defined as non-dilated left ventricular cardiomyopathy [1].

RCM characterized by restrictive cardiac physiology is caused by increased myocardial stiffness and a pathological rise in ventricular filling pressure [2]. The term restrictive derives from Latin *restrictus*, meaning tight or limiting the expansion; it is important to recognize that the term “restrictive cardiomyopathy” does not only represent a diagnostic phenotype but also describes the underlying physiology.

Since RCM is rare, clinicians involved in the diagnostic process and management of paediatric patients with RCM might not be as confident as in other more prevalent cardiomyopathies. Hence, doubts are common. Here, the most frequently asked questions posed by the initial evaluation and management of patients with RCM are addressed in a Q&A format.

## 2. How Common Is RCM?

Among the paediatric population, RCM is the least common cardiomyopathy and is typically diagnosed between the ages of 6–10 years but can present in early infancy or in adulthood, with an approximately equal sex distribution. Aetiologies vary by age and country of origin, with wide geographical differences [3]. The Pediatric Cardiomyopathy Registry in North America estimated the incidence of RCM at 0.03–0.04 per 100,000 children, accounting for approximately 4% of paediatric cardiomyopathies [4]. Meanwhile, the incidence of paediatric RCM may be higher in tropical areas of Africa, Asia and South America, where endemic forms are present [5]. The reason for an increased prevalence of RCM in the tropics is currently under scrutiny. Despite many hypotheses, the exact causes and mechanisms remain unclear. Genetics is a potential factor due to ethnic clustering, but it is difficult to tease out the confounding shared environmental exposures thought to play a role in the development of RCM in tropical areas [6]. For example, several infectious organisms have been hypothesised to elicit an exaggerated immune response [7].

## 3. Is Family History and Genetic Testing Important?

Family history of cardiomyopathy should always be investigated, as nearly 25% of RCM patients have at least one affected relative, with an even higher proportion (42%) among those with a mixed restrictive and hypertrophic phenotype [8]. This highlights the importance of a detailed family tree, genetic counselling and cascade genetic testing to identify patients at risk of developing RCM or other overlapping phenotypes.

## 4. What Are the Main Clinical Signs and Symptoms of Paediatric RCM?

Clinical manifestations range from lack of symptoms to overt heart failure (HF); the most common scenario is poor exercise tolerance and fatigue due to low cardiac output [9]. Since restrictive left ventricle (LV) cannot adjust volumes during exercise, the only way to increase output is to augment heart rate.

Symptoms of HF are nonspecific, challenging to interpret and vary with the age of onset, usually manifesting as difficulty in feeding, tachypnoea, sinus tachycardia and diaphoresis in the infant. Children with RCM frequently have a history of “recurrent lower respiratory tract infections,” wheezing or persistent cough at night [5]. Adolescence may present with fatigue, dyspnoea or abdominal pain [10].

On examination, jugular venous distention, hepatomegaly, a loud pulmonary component of second heart sound, gallop rhythm and a systolic murmur due to atrio-ventricular (AV) valve regurgitation may be present. An abnormally high respiratory rate in the newborn, puffy eyelids and sacral oedema are signs of systemic venous congestion, while ankle oedema, which is commonly seen in adults, is not found in infants [11,12].

## 5. When Should I Suspect Restrictive Phenotype on Electrocardiogram or Imaging Techniques?

**Figure 1:**
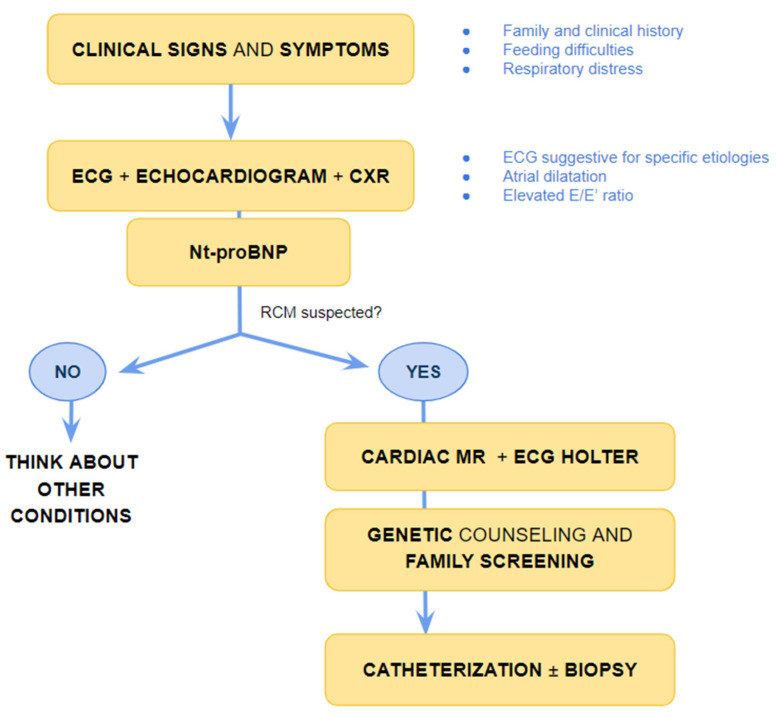
Suggested flow-chart for initial approach to patient with heart failure and suspected restrictive cardiomyopathy. CXR chest X-ray, ECG electrocardiogram; MR magnetic resonance, Nt-pro-BNP N-terminal pro B type Natriuretic Peptide, RCM-restrictive cardiomyopathy.

In the paediatric setting, diagnosing RCM at an early stage is challenging as specific paediatric guidelines for evaluating heart remodelling are missing [13]. Electrocardiogram (ECG), chest X-ray, echocardiography and NTproBNP are first line evaluations helpful in the rule-in/rule-out process for RCM diagnosis [14] (Figure 1).

**Figure 2:**
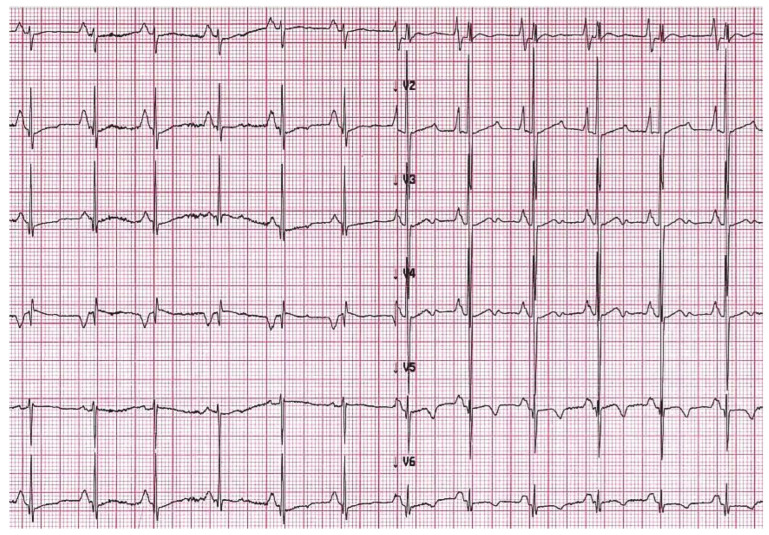
A 6-year-old boy with restrictive cardiomyopathy. Electrocardiogram shows abnormal P waves suggestive of atrial enlargement and abnormal ventricular repolarization suggestive of biventricular pressure overload.

Evidence of atrial dilatation on ECG is one of the red flags suggestive of possible RCM, emphasizing the underlying atrial remodelling (Figure 2) [15]. Atrial dilatation reflects increased filling pressures and may predispose to atrial fibrillation in adults with RCM, while this is uncommon in children. Other arrhythmias such as atrio-ventricular (AV) block or intraventricular conduction delay are rare but may develop in the context of specific aetiologies associated with RCM, as discussed below.

Echocardiographic signs reflect the increased pressure overload due to increased stiffness of the ventricles. While systolic function is commonly preserved, initial signs of diastolic dysfunction remain challenging to detect due to the lack of sensitive cutoff values in the paediatric population [13]. Tissue Doppler imaging is helpful in defining the diastolic function. In a comparison study with invasive hemodynamic measurements, the pulsed Doppler cutoff values of lateral a’ velocity ≤ 4 cm/s and pulmonary vein A wave duration of more than 156 milliseconds showed high sensitivity and specificity in detecting early RCM [16].

**Figure 3:**
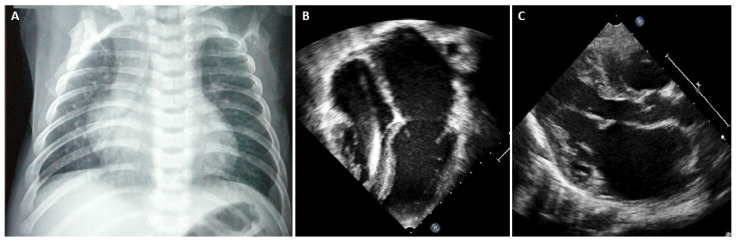
(**A**) Chest X-ray, 8-year-old boy with hypertrophic-restrictive cardiomyopathy and congestive heart failure. (**B**) Echocardiography apical 4-chambers view, 11-year-old girl with restrictive cardiomyopathy, normal ventricular volumes, preserved ejection fraction and severe atrial dilatation; we can appreciate the presence of an implantable cardiac defibrillator lead. (**C**) Echocardiography parasternal long axis view, 6-year-old boy with restrictive cardiomyopathy associated to severe left atrial dilatation.

In advanced stages of disease, atrial dilatation is common (Figure 3), pericardial effusion may develop as hemodynamic consequences of increased cardiac filling pressure and Doppler evaluation shows increased early filling velocity (E wave)/atrial filling velocity (A wave) ratio, reduced E wave deceleration time and elevated E/e’ ratio on tissue Doppler.

Chest X-ray is often helpful when HF signs are present. In overt RCM phenotype, chest radiography of patients with RCM shows cardiac enlargement due to atrial and pulmonary artery dilatation. In addition, signs of pulmonary congestion and pleural effusion might be present [17].

Cardiac magnetic resonance (CMR) can be of great importance in the diagnostic workup (Figure 1). However, the evaluation of diastolic dysfunction at CMR is still based on indirect parameters such as atrial enlargement, T1 mapping and the extension of late gadolinium enhancement (LGE) [18,19].

CMR can provide additional information regarding tissue characterization, including the extension of fibrosis, which is an important risk factor for adverse outcomes, an indirect marker of increased wall stiffness [20].

Besides the general considerations of the imaging findings of RCM, there are specific CMR findings that help in the diagnostic process for specific aetiologies and in the therapeutic management.

Moreover, CMR is helpful in guiding the clinician in differential diagnosis between RCM and constrictive pericarditis, which sometimes shows similar clinical manifestations. However, constrictive pericarditis is typically characterized by pericardial thickening, ventricular interdependence and pericardial LGE when inflammation or extensive fibrosis is present [21].

## 6. What Are the Limitations of ECG and Imaging Techniques?

Diagnosis of RCM is often challenging, especially in children. Non-invasive tests such as ECG, echocardiography and CMR can provide valuable information and are often the first steps in the diagnostic work-up of RCM, but they may have limitations that need to be considered.

ECG abnormalities might be subtle or absent in the early phase of the disease. In echocardiography, the reliability of diastolic measurements in children are challenging, given the higher heart rates and limited data on the relationships among the diastolic variables and the degree of dysfunction [22].

CMR have limitations due to its centre availability, need of sedation and the requirement to be performed in specialized centres [23].

Overall, none of the non-invasive evaluation can provide a reliable evaluation of the intracardiac pressure, while cardiac catheterization represents the gold standard for the evaluation of intracardiac pressure, the diagnosis of RCM and the reassessment of the patients during follow-up to define the management and therapeutic approach.

## 7. What Are the Aetiologies That Mainly Affect the Paediatric Population (Figure 4)?

Endomyocardial fibrosis is considered the most common cause of RCM worldwide, affecting more than 12 million people worldwide [7], mainly in tropical and subtropical areas. Endomyocardial fibrosis often affects children and young adults belonging to the poorest groups of the population [24]. It is characterized by recurrent hot phases with inflammation and eosinophilia, progressing to a chronic phase with fibrosis of the ventricular endocardium and sub-endocardium that extends from the apex upwards, often involving the AV valves. A typical CMR pattern is characterized by presence of myocardial oedema during inflammatory phases and/or associated with fibrosis [25]. The fibrous tissue markedly diminishes the volume and compliance of affected chambers determining a restrictive physiology.

Although the aetiology is unknown, several hypotheses linking infectious agents, toxins or environmental factors to the unique geographical distribution are commonly suggested as possible favouring factors.

Endocardial fibroelastosis is primarily a disease of infants and children, but can rarely present in adults as well [26]. Due to the rarity of this condition, there are not enough data on the incidence and prevalence. The usual age of presentation is the first year of life [27], and it is frequently associated with other congenital heart disease as aortic stenosis and hypoplastic left heart syndrome [28]; nonetheless, the exact aetiology is unknown.

**Figure 4:**
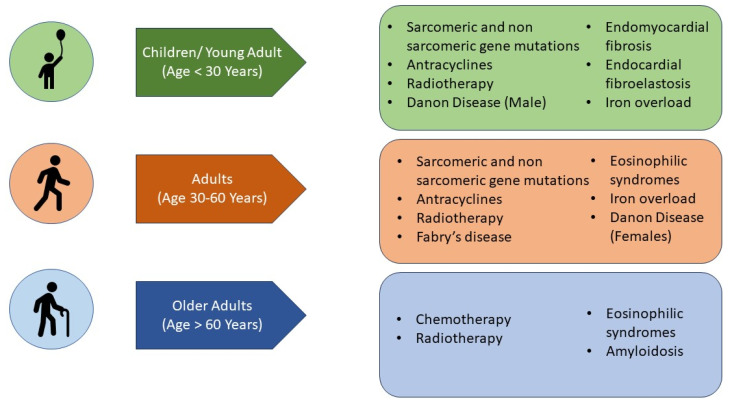
Main aetiologies of restrictive cardiomyopathy according to the age of disease presentation.

The underlying pathophysiology of endocardial fibroelastosis is the deposition of acellular fibrocartilaginous tissue in the subendothelial layer of the endocardium predominantly involving the inflow tracts and apices of ventricles [29], and intraventricular thrombi formation are an additional complication that CMR can detect [30].

## 8. What Are the Aetiologies That Can Affect Both Paediatric and Adult Populations (Figure 4)?

The Paediatric Cardiomyopathy Registry Investigators reported a pathogenic or likely pathogenic genetic variant in 50% of children with RCM, mainly in sarcomeric genes [31], also explaining possible overlapping phenotypes between HCM and RCM [31,32].

Sarcomeric gene variants in *MYBPC3* (myosin-binding protein), *MYH7* (β-myosin heavy chain), *TNNI3* (troponin I), *TNNT2* (troponin T), *TNNC1* (troponin C) [33], *ACTC1* (α-cardiac actin) [31], *TPM1* (tropomyosin 1), *MYL3* and *MYL2* (myosin light chain) [34] have been associated with both HCM and RCM [4,35,36]. All these genes are involved in the contractile apparatus of the heart and mutations in these proteins modify sarcomere contractility and ion channel function and lead to impaired relaxation of myocytes determining restrictive physiology.

Patients with gene variants involving thin filaments, such as troponin, may develop hypertrophic ventricular remodelling associated with an increased amount of interstitial fibrosis and myocardial disarray, which often affects ventricle compliance regardless of wall thickness.

Titin, encoded by the gene *TTN*, is the largest human protein [37] and provides architectural support in sarcomers [38]. *TTN* variants are related to muscular dystrophy and different phenotypes of cardiomyopathies. Truncations of *TTN* are the most frequent in DCM and rarely have been implicated in the pathogenesis of HCM, ACM and RCM [39,40]. A large number of missense and truncation variants of *TTN* have also been reported in the general population [41]. Thus, the clinical interpretation of *TTN* variants remains a challenge.

Non-sarcomeric gene variants share a wide heterogeneity in clinical presentation and, particularly, the possibility to determine RCM associated with skeletal muscle involvement and arrhythmias.

One of the genes involved in the development of RCM is *MYPN* (encoding myopalladin). The mechanism proposed for development of RCM is altered myofibrillogenesis and consequent myofibrillar disarray and restrictive physiology [42]. However, only a few case reports have been described in the paediatric population.

In 1998, a *DES* variant was described as associated with RCM for the first time [43]. Clinical phenotypes associated with *DES* variants are broad and include DCM, HCM and ACM. Different mutations might be causative of RCM; an example of this is *DES* c.735G>C causing a skipping of the third *DES* exon determining the expression of truncated desmin, leaving it unable to form regular intermediate filaments and being associated to RCM and atrial fibrillation [44]. Meanwhile, *DES* gene variant (c.364T>C) was described in a family with RCM and AV block [45].

Filamin-C is an actin cross-linking protein expressed in the heart and skeletal muscle. *FLNC* variants present a wide spectrum of clinical presentation and the first description of its association with RMC was in 2016 [46]. However, overlapping phenotypes can coexist. An example of this is ROD2 domain filamin C missense mutations that exhibit a distinctive cardiac phenotype with RCM/HCM and saw-tooth cardiomyopathy [47,48]. *FLNC* missense variants can cause early-onset RCM associated with variable degrees of skeletal myopathy, combined with mild CK elevation and supraventricular arrhythmias, whereas *FLNC* truncating variants have been found in patients with an ACM/DCM phenotype characterized by a high risk of ventricular arrhythmias [48].

Filamin-C phenotype is often characterized by low QRS voltage and T waves inversion in the inferior-lateral leads [49]. Analysing CMR features, a characteristic subepicardial “ring-like” fibrotic pattern is common [50].

In 2017 a new missense variant c.326A>G in *CRYAB* gene encoding αB-crystallin was described. It is a member of the small heat shock protein family, which is highly expressed in cardiac and skeletal muscle. This variant was reported in small family with RCM in combination with skeletal myopathy with an early onset of the disease [51].

Danon disease is a rare X-linked–dominant storage disease due to mutations in the *LAMP2* gene affecting lysosomal degradation of glycogen [52,53]. The diagnosis is suggested by clinical history and possibly by the finding of glycogen deposits in a skeletal muscle biopsy. Danon disease typically affects adolescent males with a triad of cardiomyopathy, skeletal myopathy and mental impairment. Females can be affected, although usually later in life and with a milder expression of the disease. In Danon disease, like other metabolic cardiomyopathies, one of the most common ECG findings is ventricular pre-excitation. Other common ECG abnormalities include high precordial R and S wave voltages with deep T waves inversion, atrial fibrillation, AV block and ventricular arrhythmias [54]. Cardiomyopathy usually expresses as HCM, DCM and, in a few cases, RCM. The vast majority of patients rapidly evolve into progressive HF. At CMR, myocardial mid-wall fibrosis starting from the inferior and lateral wall of the LV with a characteristic sparing of mid-interventricular septum is usually seen.

Iron overload is a rare condition that may occur in primary hemochromatosis, an autosomal-recessive condition caused by mutations in various proteins involved in iron metabolism [55], or in secondary forms of iron overload including hereditary anaemias (e.g., sickle cell anaemia and α- or β-thalassemia), multiple blood transfusions, myelodysplastic syndromes and end-stage renal disease [55].

When the amount of circulating iron exceed the binding capacity of transferrin, the excess of iron increases free-radical species leading to oxidative damage of lipid cell membranes, nucleic acids and calcium cycling proteins in cardiomyocytes [56,57].

Untreated iron-overload in children and young adults may eventually lead to impaired diastolic function determining RCM [58].

While echocardiography findings cannot predict the amount of iron deposition [59], CMR represent the main imaging technique for early diagnosis and quantify myocardial and hepatic iron deposition using specific gradient echo sequences in T2* relaxation time. A myocardium T2* time value < 20 ms is indicative of myocardial siderosis and a T2* value <10 ms is considered diagnostic for severe iron overload.

Anthracyclines are the most common cause of chemotherapy-induced cardiotoxicity with varying clinical presentations, including acute, early onset and late-onset (more than one year after chemotherapy). Although cardiotoxicity can occur with any dose of anthracycline, cumulative doses of above 250 mg/m^2^ represent a predisposing factor [60]. In a study from Trachtenberg et al., among paediatric patients managed with anthracycline who developed DCM, frequently progressed to restrictive physiology [61]. These findings were more likely in individuals at younger age at cancer diagnosis and longer follow-up [62,63]. A hypothesis is that a defined number of myocytes are present in the heart, and subsequent myocardial growth occurs by increasing the size of the myocytes. In younger patients, the cardiotoxic effect of chemotherapy might impair the ability of the heart to generate an average adult myocardial mass [64]. Inadequate increase in ventricular mass in relation to somatic growth results in restrictive physiology [65].

Therefore, cardiotoxicity due to anthracyclines in children may differ from that in adults. Cardiotoxicity most commonly manifests in adults as acquired DCM, while in children it initially manifests as DCM, and in some cases, remodelling progresses to RCM with somatic growth.

Radiotherapy is frequently used together with surgery/chemotherapy in thoracic malignancies and lymphomas. The clinical manifestations of myocardial injury caused by radiotherapy generally occur after many years from treatment [66] and they mainly manifest as fibrosis of the myocardium and pericardium [67]. The risk of radiation-mediated RCM is especially high for patients who have received a high dose of chest radiation and/or concomitant treatment with cardiotoxic chemotherapy [68].

Time- and radiation dose-dependent inflammation activates fibrogenic pathways [69,70]. Fibrosis of the interstitium and replacement fibrosis of dead myocytes [71] can lead to a RCM. The right ventricle is more often affected than the LV given its more anterior and closer position to the radiation beam [72]. Similarly, fibrotic deposition in the pericardium can lead to constrictive pericarditis.

An important aspect for both chest radiotherapy and anthracycline therapy is to continue surveillance and imaging monitoring over time, as cardiotoxicity can develop years after the end of oncological treatment.

## 9. Which Causes of Adult RCM May Be Identified (Preclinically) in the Paediatric Age (Figure 4)?

Anderson-Fabry disease is the most common glycogen storage disorder. It is an X-linked–recessive sphingolipidosis caused by α-galactosidase-A deficiency. Accumulation of globotriaosylceramide in the lysosomes occurs in various tissues and organs, including the heart, kidneys, vasculature and peripheral nervous system [73,74,75].

Extracardiac manifestations of the disease may occur in childhood. The first symptoms generally include chronic neuropathic pain, episodic severe pain crises, hypohidrosis, angiokeratomas, gastrointestinal disturbances and corneal opacity (cornea verticillata). Occult kidney injury may occur at a young age, including albuminuria and glomerulosclerosis, while overt renal failure, cardiac involvement and strokes occurs in adulthood. Cardiac manifestations are generally reported in men aged older than 30 years and women aged older than 40 years [76], leading to progressive hypertrophy and diastolic dysfunction [77,78].

Eosinophilic syndrome with cardiac involvement was first described in 1936 by Wilhelm Löffler. This condition is rare in children and occurs in the presence of eosinophilic infiltration of cardiac tissues. The severity of cardiac injuries is related to the duration and to the intensity of eosinophilia [79], particularly in cases of profound eosinophilia [80], that can be a clue of the disease. Eosinophilic infiltration into the tissues leads to the typical complications: intracardiac thrombi and fibrotic process involving the myocardium and valves resulting in RCM [81,82,83].

## 10. What Are the Main Haemodynamic Features?

Right heart catheterization is an invasive haemodynamic procedure that allows direct measurement of right-sided cardiac pressures and calculation of cardiac output.

Normally, the right atrial pressure curve exhibits five deflections, three positive (a, c and v) and two negative (x and y): the “a” wave is a diastolic wave and is generated by atrial contraction; the “c” wave is produced by the protrusion of the AV valve into the atrial cavity when it is closed during the isometric contraction of the ventricle; the “x” wave results from the pressure drop in the atrial cavity induced by ventricular systole; the “v” wave (typically very high) is produced by the increased atrial pressure due to atrial filling while the AV valve is still closed during ventricular systole; and the “y” wave arises from the pressure drop induced by the opening of the AV valve, allowing atrial emptying and rapid ventricular filling.

In RCM, the haemodynamic profile is characterized by elevated diastolic filling pressures and a rapid equalization of filling pressures among the four cardiac chambers during diastole, resulting in a prominent “y” descent wave and the appearance of the “square root” or “dip and plateau” sign on ventricular pressure curves (characterized by an early decrease in ventricular diastolic pressure followed by a rapid rise to a plateau phase).

While many of these findings are also observed in constrictive pericarditis (CP), there are several differences: in RCM. LV end-diastolic pressures are higher than those in the right ventricle, and there is no evidence of ventricular interdependence with parallel changes in LV and right ventricular pressure curve areas. Additionally, there is little respiratory variation in flow or pressure.

## 11. When Endomyocardial Biopsy Is Useful?

In the context of RCM, the role of endomyocardial biopsy (EMB) remains controversial, both for adult and paediatric populations, and the combination of genetic analysis and imaging techniques has debunked the role of EMB in the diagnostic work-up of RCM. In the majority of cases, EBM shows non-specific findings, such as myocyte hypertrophy and interstitial and/or endocardial fibrosis.

However, due to the possibility of treatable disorders, EMB may reveal a specific infiltrative disorder hemochromatosis, myocardial fibrosis and disarray and amyloid fibrils consistent with specific aetiologies.

In a 2007 scientific statement by the American Heart Association, the American College of Cardiology and the European Society of Cardiology, EMB was indicated in the setting of HF associated with unexplained restrictive cardiomyopathy (Class of Recommendation IIa, Level of Evidence C) without distinguishing between adults and children [84].

More recently, in the 2022 position paper of the Italian Society of Cardiology (SIC) and the Italian Society of Paediatric Cardiology (SICP), EMB was suggested only for the adult population when non-invasive tests have not provided conclusive results [85].

It should be emphasized that in the paediatric population, the EMB procedure is more complex and generally requires general anaesthesia [86].

While the rate of major complications in the adult population is very low, below 1%, in children the incidence of adverse events is higher, ranging from 9.7% to 15.5% [87,88,89], and severe complications occur in 2.9% to 5.2% of cases [87,90].

In conclusion, in the paediatric context, EMB may be considered only in cases where non-invasive diagnostic methods have not yielded a definitive diagnosis, with careful consideration of the risk–benefit ratio.

## 12. What Is the Prognosis of RCM?

RCM usually has a poor outcome with a high mortality due to progressive HF [91]. It has been reported that after 5 years from the diagnosis, the cumulative incidence of heart transplantation (HT) was 58% in RCM patients with pure restrictive phenotype and 30% in those with a mixed hypertrophic and restrictive phenotype, with no significant difference in the mortality rate [8].

In a Spanish case series, an even worse prognosis has been documented, with only 20% of patients with RCM alive and free from transplantation at 5 years from the diagnosis [92].

Identifying the exact risk of HF in paediatric RCM is challenging because of the low prevalence of RCM and the heterogeneous underlying aetiology. Overt HF is described in approximately 45% of paediatric patients with RCM [91,93], while New York Heart Association (NYHA) functional classes III and IV have been documented in nearly 25% of paediatric RCM [91]. Advanced NYHA classes and a need for multiple hospitalizations have been identified as the main predictors for adverse outcomes [93], specifically for HT and all-cause mortality [91,93].

Because of the suggested correlation with increased mortality, signs and symptoms of HF should be routinely investigated to promptly refer patients for advanced HF treatment.

However, PH can be more subtle to identify, even if it is a common complication in RCM and is associated with worse prognosis. Therefore, the presence of PH should be periodically investigated after performing invasive cardiac catheterism to exclude the development of an irreversible pulmonary vascular remodelling that contraindicates HT.

Conversely, the risk of sudden death is less clearly defined as only small cohorts of paediatric patients with RCM have been under scrutiny for arrhythmic risk stratification, and risk of arrhythmias is influenced by the underlying aetiology [94].

## 13. What Pharmacological Treatments Are Available for the Most Common Forms?

Baseline treatment for symptoms relief in paediatric RCM consists in diuretic therapy; however, disease-modifying therapies are available for Danon, Fabry, iron overload cardiomyopathy, eosinophilic syndromes, HCM and amyloidosis.

Diuretics can improve symptoms and systemic venous congestion, but excess use will reduce preload and hence cardiac output [4]. Diuretic therapy is recommended in symptomatic patients with monitoring of renal function and blood pressure.

Recently, SGLT2 inhibitors demonstrated their beneficial effects on renal and HF in adult population and they have been approved for HF both with and without preserved systolic function. Preliminary data supporting their use in paediatric population are emerging and demonstrate their safety in this population [95].

Conversely, considering the peculiar pathophysiology of RCM, the routine use of betablockers or non-dihydropyridine calcium channel blockers should be generally avoided. The restrictive physiology of RCM does not allow an increase in stroke volume and adequate cardiac output is often ensured by an increased heart rate. Thus, treatments with a negative chronotropic effect are generally poorly-tolerated. Therfore, heart rate should be maintained as high as necessary for adequate systemic perfusion. Furthermore, in specific aetiologies such as Danon disease, they may elicit the evolution into advanced AV blocks.

In few cases, RCM coexists with systemic arterial hypertension. In these patients, angiotensin converting enzyme inhibitors or non-dihydropyridine calcium channel blockers may be considered [96].

Finally, thromboembolic events are possible in RCM and an accurate evaluation for anti-coagulation therapy is recommended as a secondary and sometimes a primary prevention [97].

Despite a careful follow-up and tailored treatment, progressive HF in RCM may lead patients to advanced HF symptoms requiring referral for HT or mechanical circulatory support (MCS).

## 14. When Should I Refer Patients for Cardiac Hemodynamic Mechanical Support or Heart Transplantation?

Paediatric RCM carries a poor prognosis. The primary cause of death is progressive HF [8], and the median survival after diagnosis is approximately 2 years [17,94]. Due to the unfavourable prognosis, cardiac transplantation has been proposed as the preferred treatment for this patient population.

There are several red flags (pulmonary congestion, increased pulmonary pressure, severe left atrial dilation and reduction in ventricular contractility) suggesting a need for refer patients to a tertiary centre for HT evaluation. However, the optimal timing and criteria for listing RCM patients for HT are still not well defined [98].

It has been observed that the mortality while on the waiting list for children with RCM is relatively low. This is likely due to a common practice of listing RCM patients shortly after diagnosis, given the acknowledged poor outcome of the disease and limited success of medical therapy [99].

Outcomes for paediatric HT in patients with RCM have been documented only by a few reports: thirty-day survival was around 94–96% [100,101] and 1-year survival was around 82–86% [100,102].

Despite HT being is a possible solution, the shortage of donor organs has become a significant concern. As a result, surgical alternatives such as MCS are gaining importance as a bridge to transplantation [103].

The main indication for MCS is low cardiac output syndrome, suggested by the presence of rapid circulatory deterioration with a cardiac index <2.0 L/min/m^2^ and/or dependence on inotropes, mixed venous saturation of <40%; oliguria (<1 mL/kg/min) and critical peripheral perfusion.

However, RCM has been considered a risk factor for poor outcomes in MCS [91], especially in children under 3 years of age and weighing less than 10 kg [104]. This is due to diastolic HF and a small LV cavity, leading to an increased risk of persistent right HF, thromboembolism and insufficient LV drainage with consequent poor pump performance [103].

Therefore, novel modifications to LV cannulation techniques are being explored, such as trans-septal left atrium-to-aorta ventricular assist device cannulation, atrial cannulation or biventricular support [3,105,106].

The main concern in patients with RCM is the presence of PH (in RCM the prevalence of PH is up to 50% at diagnosis) [101,107], as PH correlates with a higher incidence of postoperative right ventricular failure [103]. For these reasons, pulmonary vascular resistance index (PVRI) greater than 6 Wood Units (W.U.) × m^2^, trans pulmonary gradient ≥15 mmHg and pulmonary diastolic gradient >7 mmHg are considered a contraindication to cardiac transplant [98,101,108].

**Figure 5:**
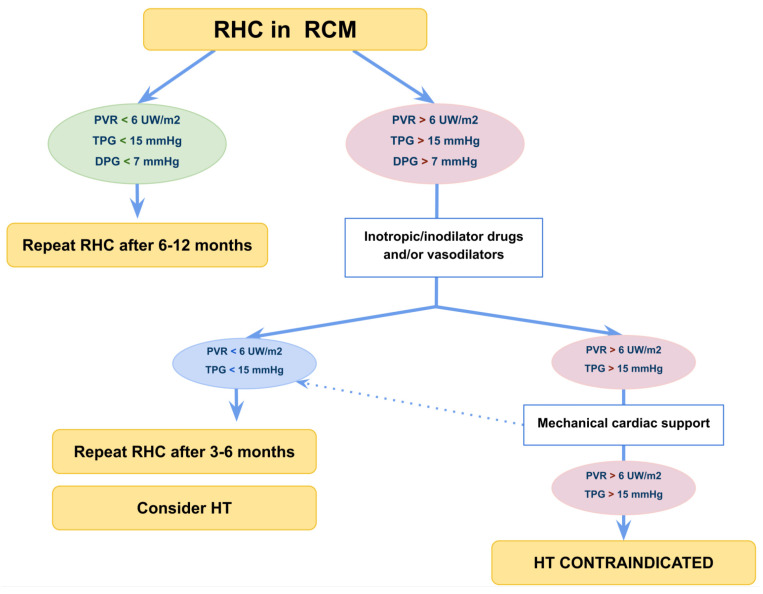
Flow-chart management of paediatric restrictive cardiomyopathy). Right heart catheterization is usually indicated for all patients referred for HT. However, it is useful to stratify prognosis in patients in NYHA class I to II since severe PH and low cardiac output can develop with mild or subtle symptoms. DPG, diastolic pulmonary gradient; HT, heart transplantation; PVR, pulmonary vascular resistance; RHC, right heart catheterization; TPG, transpulmonary gradient.

Recent data from children awaiting heart transplant show that those with an PVRI >6 W.U. m^2^ had better waitlist outcomes when placed on MCS compared to those who were not [109,110,111,112], leading long-term MCS implantation to be considered in advanced stages of the disease as a bridge to HT by improving haemodynamics and reducing post-capillary PH. Figure 5 shows the management of paediatric RCM base on right heart catheterization [5].

## 15. Conclusions

RCM is a rare clinical condition in the paediatric age, the low prevalence of this cardiomyopathy and the heterogeneity of the potential underlying aetiology explain the difficulty in finding a common management for RCM. However, there are few common characteristics of the disease: the clinical suspect requires invasive cardiac catheterization for diagnosis; in Western countries, a considerable proportion of paediatric patients with RCM present sarcomeric or non-sarcomeric gene variants. A family history of cardiomyopathy is common, underlining the importance of genetic counselling [113].

Unfortunately, prognosis of paediatric RCM is often ominous; thus, we support an early referral to tertiary centres for advance HF treatment.

Recently, specific drug treatments have been developed for HF with preserved ejection fractions and for several genetic and non-genetic causes of RCM. In many cases, these treatments are under scrutiny in paediatric patients and may change the natural history of the disease, redefining the clinical scenario of paediatric RCM in the near future.

## References

[1] Arbelo E., Protonotarios A., Gimeno J.R., Arbustini E., Barriales-Villa R., Basso C., Bezzina C.R., Biagini E., Blom N.A., De Boer R.A. (2023). 2023 ESC Guidelines for the management of cardiomyopathies. Eur. Heart J..

[2] Rapezzi C., Aimo A., Barison A., Emdin M., Porcari A., Linhart A., Keren A., Merlo M., Sinagra G. (2022). Restrictive cardiomyopathy: Definition and diagnosis. Eur. Heart J..

[3] Denfield S.W. (2021). Overview of pediatric restrictive cardiomyopathy—2021. Prog. Pediatr. Cardiol..

[4] Lee T.M., Hsu D.T., Kantor P., Towbin J.A., Ware S.M., Colan S.D., Chung W.K., Jefferies J.L., Rossano J.W., Castleberry C.D. (2017). Pediatric Cardiomyopathies. Circ. Res..

[5] Ditaranto R., Caponetti A.G., Ferrara V., Parisi V., Minnucci M., Chiti C., Baldassarre R., Di Nicola F., Bonetti S., Hasan T. (2021). Pediatric Restrictive Cardiomyopathies. Front. Pediatr..

[6] Mocumbi A.O., Ferreira M.B., Sidi D., Yacoub M.H. (2008). A population study of endomyocardial fibrosis in a rural area of Mozambique. N. Engl. J. Med..

[7] Bukhman G., Ziegler J., Parry E. (2008). Endomyocardial Fibrosis: Still a Mystery after 60 Years. PLoS Negl. Trop. Dis..

[8] Webber S.A., Lipshultz S.E., Sleeper L.A., Lu M., Wilkinson J.D., Addonizio L.J., Canter C.E., Colan S.D., Everitt M.D., Jefferies J.L. (2012). Outcomes of restrictive cardiomyopathy in childhood and the influence of phenotype: A report from the Pediatric Cardiomyopathy Registry. Circulation.

[9] Muchtar E., Blauwet L.A., Gertz M.A. (2017). Restrictive Cardiomyopathy: Genetics, Pathogenesis, Clinical Manifestations, Diagnosis, and Therapy. Circ. Res..

[10] Masarone D., Valente F., Rubino M., Vastarella R., Gravino R., Rea A., Russo M.G., Pacileo G., Limongelli G. (2017). Pediatric Heart Failure: A Practical Guide to Diagnosis and Management. Pediatr. Neonatol..

[11] Lipshultz S.E., Cochran T.R., Briston D.A., Brown S.R., Sambatakos P.J., Miller T.L., Carrillo A.A., Corcia L., Sanchez J.E., Diamond M.B. (2013). Pediatric cardiomyopathies: Causes, epidemiology, clinical course, preventive strategies and therapies. Future Cardiol..

[12] Quennelle S., Bonnet D. (2023). Pediatric heart failure with preserved ejection fraction, a review. Front. Pediatr..

[13] Sasaki N., Garcia M., Ko H.H., Sharma S., Parness I.A., Srivastava S. (2015). Applicability of published guidelines for assessment of left ventricular diastolic function in adults to children with restrictive cardiomyopathy: An observational study. Pediatr. Cardiol..

[14] Barretto A.C., Mady C., Nussbacher A., Ianni B.M., Oliveira S.A., Jatene A., Ramires J.A. (1998). Atrial fibrillation in endomyocardial fibrosis is a marker of worse prognosis. Int. J. Cardiol..

[15] Muraji S., Sumitomo N., Imamura T., Yasuda K., Nishihara E., Iwamoto M., Tateno S., Doi S., Hata T., Kogaki S. (2021). Diagnostic value of P-waves in children with idiopathic restrictive cardiomyopathy. Heart Vessel..

[16] Ryan T.D., Madueme P.C., Jefferies J.L., Michelfelder E.C., Towbin J.A., Woo J.G., Sahay R.D., King E.C., Brown R., Moore R.A. (2017). Utility of Echocardiography in the Assessment of Left Ventricular Diastolic Function and Restrictive Physiology in Children and Young Adults with Restrictive Cardiomyopathy: A Comparative Echocardiography-Catheterization Study. Pediatr. Cardiol..

[17] Denfield S.W., Webber S.A. (2010). Restrictive Cardiomyopathy in Childhood. Heart Fail. Clin..

[18] Rosales X.Q., Moser S.J., Tran T., McCarthy B., Dunn N., Habib P., Simonetti O.P., Mendell J.R., Raman S.V. (2011). Cardiovascular magnetic resonance of cardiomyopathy in limb girdle muscular dystrophy 2B and 2I. J. Cardiovasc. Magn. Reson..

[19] de Ville de Goyet M., Brichard B., Robert A., Renard L., Veyckemans F., Vanhoutte L., Moniotte S. (2015). Prospective cardiac MRI for the analysis of biventricular function in children undergoing cancer treatments. Pediatr. Blood Cancer.

[20] Axelsson Raja A., Farhad H., Valente A.M., Couce J.-P., Jefferies J.L., Bundgaard H., Zahka K., Lever H., Murphy A.M., Ashley E. (2018). Prevalence and Progression of Late Gadolinium Enhancement in Children and Adolescents With Hypertrophic Cardiomyopathy. Circulation.

[21] Garcia M.J. (2016). Constrictive Pericarditis Versus Restrictive Cardiomyopathy?. J. Am. Coll. Cardiol..

[22] Lipshultz S.E., Law Y.M., Asante-Korang A., Austin E.D., Dipchand A.I., Everitt M.D., Hsu D.T., Lin K.Y., Price J.F., Wilkinson J.D. (2019). Cardiomyopathy in Children: Classification and Diagnosis: A Scientific Statement From the American Heart Association. Circulation.

[23] Habib G., Bucciarelli-Ducci C., Caforio A.L.P., Cardim N., Charron P., Cosyns B., Dehaene A., Derumeaux G., Donal E., Dweck M.R. (2017). Multimodality Imaging in Restrictive Cardiomyopathies: An EACVI expert consensus document In collaboration with the “Working Group on myocardial and pericardial diseases” of the European Society of Cardiology Endorsed by The Indian Academy of Echocardiography. Eur. Heart J. Cardiovasc. Imaging.

[24] Rutakingirwa M., Ziegler J.L., Newton R., Freers J. (1999). Poverty and eosinophilia are risk factors for endomyocardial fibrosis (EMF) in Uganda. Trop. Med. Int. Health.

[25] de Carvalho F.P., Azevedo C.F. (2020). Comprehensive Assessment of Endomyocardial Fibrosis with Cardiac MRI: Morphology, Function, and Tissue Characterization. Radiographics.

[26] Ozdemir D., Cortopassi I.O., McNamara R.L. (2019). An illustrative case of endocardial fibroelastosis and recalcitrant intracardiac thrombosis: A case report. Thromb. J..

[27] Harris L.C., Nghiem Q.X. (1972). Cardiomyopathies in infants and children. Prog. Cardiovasc. Dis..

[28] Weixler V., Hammer P.E., Marx G.R., Emani S.M., Del Nido P.J., Friehs I. (2019). Flow disturbances and progression of endocardial fibroelastosis—A case report. Cardiovasc. Pathol..

[29] Schryer M.J., Karnauchow P.N. (1974). Endocardial fibroelastosis; etiologic and pathogenetic considerations in children. Am. Heart J..

[30] Lane K.L., Herzberg A.J., Reimer K.A., Bradford W.D., Schall S.A. (1991). Endocardial fibroelastosis with coronary artery thromboembolus and myocardial infarction. Clin. Pediatr..

[31] Kaski J.P., Syrris P., Burch M., Tome-Esteban M.-T., Fenton M., Christiansen M., Andersen P.S., Sebire N., Ashworth M., Deanfield J.E. (2008). Idiopathic restrictive cardiomyopathy in children is caused by mutations in cardiac sarcomere protein genes. Heart.

[32] Arbustini E., Pasotti M., Pilotto A., Pellegrini C., Grasso M., Previtali S., Repetto A., Bellini O., Azan G., Scaffino M. (2006). Desmin accumulation restrictive cardiomyopathy and atrioventricular block associated with desmin gene defects. Eur. J. Heart Fail..

[33] Ploski R., Rydzanicz M., Ksiazczyk T.M., Franaszczyk M., Pollak A., Kosinska J., Michalak E., Stawinski P., Ziolkowska L., Bilinska Z.T. (2016). Evidence for troponin C (*TNNC1*) as a gene for autosomal recessive restrictive cardiomyopathy with fatal outcome in infancy. Am. J. Med. Genet..

[34] Caleshu C., Sakhuja R., Nussbaum R.L., Schiller N.B., Ursell P.C., Eng C., De Marco T., McGlothlin D., Burchard E.G., Rame J.E. (2011). Furthering the link between the sarcomere and primary cardiomyopathies: Restrictive cardiomyopathy associated with multiple mutations in genes previously associated with hypertrophic or dilated cardiomyopathy. Am. J. Med. Genet..

[35] Pinto J.R., Parvatiyar M.S., Jones M.A., Liang J., Potter J.D. (2008). A Troponin T Mutation That Causes Infantile Restrictive Cardiomyopathy Increases Ca2+ Sensitivity of Force Development and Impairs the Inhibitory Properties of Troponin. J. Biol. Chem..

[36] Ware S., Quinn M., Ballard E., Miller E., Uzark K., Spicer R. (2007). Pediatric restrictive cardiomyopathy associated with a mutation in β-myosin heavy chain. Clin. Genet..

[37] Bang M.-L., Centner T., Fornoff F., Geach A.J., Gotthardt M., McNabb M., Witt C.C., Labeit D., Gregorio C.C., Granzier H. (2001). The Complete Gene Sequence of Titin, Expression of an Unusual ≈700-kDa Titin Isoform, and Its Interaction With Obscurin Identify a Novel Z-Line to I-Band Linking System. Circ. Res..

[38] Linke W.A., Hamdani N. (2014). Gigantic Business: Titin Properties and Function Through Thick and Thin. Circ. Res..

[39] Gigli M., Begay R.L., Morea G., Graw S.L., Sinagra G., Taylor M.R.G., Granzier H., Mestroni L. (2016). A Review of the Giant Protein Titin in Clinical Molecular Diagnostics of Cardiomyopathies. Front. Cardiovasc. Med..

[40] Peled Y., Gramlich M., Yoskovitz G., Feinberg M.S., Afek A., Polak-Charcon S., Pras E., Sela B.-A., Konen E., Weissbrod O. (2014). Titin Mutation in Familial Restrictive Cardiomyopathy. Int. J. Cardiol..

[41] Herman D.S., Lam L., Taylor M.R.G., Wang L., Teekakirikul P., Christodoulou D., Conner L., DePalma S.R., McDonough B., Sparks E. (2012). Truncations of titin causing dilated cardiomyopathy. N. Engl. J. Med..

[42] Purevjav E., Arimura T., Augustin S., Huby A.-C., Takagi K., Nunoda S., Kearney D.L., Taylor M.D., Terasaki F., Bos J.M. (2012). Molecular basis for clinical heterogeneity in inherited cardiomyopathies due to myopalladin mutations. Hum. Mol. Genet..

[43] Goldfarb L.G., Park K.-Y., Cervenáková L., Gorokhova S., Lee H.-S., Vasconcelos O., Nagle J.W., Semino-Mora C., Sivakumar K., Dalakas M.C. (1998). Missense mutations in desmin associated with familial cardiac and skeletal myopathy. Nat. Genet..

[44] Brodehl A., Hain C., Flottmann F., Ratnavadivel S., Gaertner A., Klauke B., Kalinowski J., Körperich H., Gummert J., Paluszkiewicz L. (2021). The Desmin Mutation DES-c.735G>C Causes Severe Restrictive Cardiomyopathy by Inducing In-Frame Skipping of Exon-3. Biomedicines.

[45] Brodehl A., Pour Hakimi S.A., Stanasiuk C., Ratnavadivel S., Hendig D., Gaertner A., Gerull B., Gummert J., Paluszkiewicz L., Milting H. (2019). Restrictive Cardiomyopathy is Caused by a Novel Homozygous Desmin (DES) Mutation p.Y122H Leading to a Severe Filament Assembly Defect. Genes.

[46] Brodehl A., Ferrier R.A., Hamilton S.J., Greenway S.C., Brundler M.-A., Yu W., Gibson W.T., McKinnon M.L., McGillivray B., Alvarez N. (2016). Mutations in FLNC are Associated with Familial Restrictive Cardiomyopathy. Hum. Mutat..

[47] Bermúdez-Jiménez F.J., Carriel V., Santos-Mateo J.J., Fernández A., García-Hernández S., Ramos K.A., Piqueras-Flores J., Cabrera-Romero E., Barriales-Villa R., de la Higuera Romero L. (2023). ROD2 domain filamin C missense mutations exhibit a distinctive cardiac phenotype with restrictive/hypertrophic cardiomyopathy and saw-tooth myocardium. Rev. Esp Cardiol..

[48] Girolami F., Passantino S., Ballerini A., Gozzini A., Porcedda G., Olivotto I., Favilli S. (2022). Clinical Exome Sequencing Revealed a De Novo FLNC Mutation in a Child with Restrictive Cardiomyopathy. Cardiogenetics.

[49] Celeghin R., Cipriani A., Bariani R., Bueno Marinas M., Cason M., Bevilacqua M., De Gaspari M., Rizzo S., Rigato I., Da Pozzo S. (2022). Filamin-C variant-associated cardiomyopathy: A pooled analysis of individual patient data to evaluate the clinical profile and risk of sudden cardiac death. Heart Rhythm..

[50] Gigli M., Stolfo D., Graw S.L., Merlo M., Gregorio C., Nee Chen S., Dal Ferro M., PaldinoMD A., De Angelis G., Brun F. (2021). Phenotypic Expression, Natural History, and Risk Stratification of Cardiomyopathy Caused by Filamin C Truncating Variants. Circulation.

[51] Brodehl A., Gaertner-Rommel A., Klauke B., Grewe S.A., Schirmer I., Peterschröder A., Faber L., Vorgerd M., Gummert J., Anselmetti D. (2017). The novel αB-crystallin (CRYAB) mutation p.D109G causes restrictive cardiomyopathy. Hum. Mutat..

[52] Nishino I., Fu J., Tanji K., Yamada T., Shimojo S., Koori T., Mora M., Riggs J.E., Oh S.J., Koga Y. (2000). Primary LAMP-2 deficiency causes X-linked vacuolar cardiomyopathy and myopathy (Danon disease). Nature.

[53] Arad M., Maron B.J., Gorham J.M., Johnson W.H., Saul J.P., Perez-Atayde A.R., Spirito P., Wright G.B., Kanter R.J., Seidman C.E. (2005). Glycogen Storage Diseases Presenting as Hypertrophic Cardiomyopathy. N. Engl. J. Med..

[54] Rigolli M., Kahn A.M., Brambatti M., Contijoch F.J., Adler E.D. (2021). Cardiac Magnetic Resonance Imaging in Danon Disease Cardiomyopathy. JACC Cardiovasc. Imaging.

[55] Murphy C.J., Oudit G.Y. (2010). Iron-Overload Cardiomyopathy: Pathophysiology, Diagnosis, and Treatment. J. Card. Fail..

[56] Khamseekaew J., Kumfu S., Chattipakorn S.C., Chattipakorn N. (2016). Effects of Iron Overload on Cardiac Calcium Regulation: Translational Insights Into Mechanisms and Management of a Global Epidemic. Can. J. Cardiol..

[57] Walter P.B., Fung E.B., Killilea D.W., Jiang Q., Hudes M., Madden J., Porter J., Evans P., Vichinsky E., Harmatz P. (2006). Oxidative stress and inflammation in iron-overloaded patients with ?-thalassaemia or sickle cell disease. Br. J. Haematol..

[58] Oudit G.Y., Trivieri M.G., Khaper N., Liu P.P., Backx P.H. (2006). Role of L-type Ca^2+^ channels in iron transport and iron-overload cardiomyopathy. J. Mol. Med..

[59] Kremastinos D.T., Farmakis D., Aessopos A., Hahalis G., Hamodraka E., Tsiapras D., Keren A. (2010). Beta-thalassemia cardiomyopathy: History, present considerations, and future perspectives. Circ. Heart Fail..

[60] Lipshultz S.E., Scully R.E., Stevenson K.E., Franco V.I., Neuberg D.S., Colan S.D., Silverman L.B., Moslehi J.J., Cheng S., Sallan S.E. (2014). Hearts too small for body size after doxorubicin for childhood ALL: Grinch syndrome. JCO.

[61] Trachtenberg B.H., Landy D.C., Franco V.I., Henkel J.M., Pearson E.J., Miller T.L., Lipshultz S.E. (2011). Anthracycline-Associated Cardiotoxicity in Survivors of Childhood Cancer. Pediatr. Cardiol..

[62] Lipshultz S.E., Lipsitz S.R., Sallan S.E., Dalton V.M., Mone S.M., Gelber R.D., Colan S.D. (2005). Chronic progressive cardiac dysfunction years after doxorubicin therapy for childhood acute lymphoblastic leukemia. J. Clin. Oncol..

[63] Bansal N., Amdani S., Lipshultz E.R., Lipshultz S.E. (2017). Chemotherapy-induced cardiotoxicity in children. Expert. Opin. Drug Metab. Toxicol..

[64] Kamphuis J.A.M., Linschoten M., Cramer M.J., Doevendans P.A., Asselbergs F.W., Teske A.J. (2020). Early- and late anthracycline-induced cardiac dysfunction: Echocardiographic characterization and response to heart failure therapy. Cardiooncology.

[65] Lipshultz S.E., Franco V.I., Miller T.L., Colan S.D., Sallan S.E. (2015). Cardiovascular Disease in Adult Survivors of Childhood Cancer. Annu. Rev. Med..

[66] Heidenreich P.A., Hancock S.L., Lee B.K., Mariscal C.S., Schnittger I. (2003). Asymptomatic cardiac disease following mediastinal irradiation. J. Am. Coll. Cardiol..

[67] Darby S.C., Cutter D.J., Boerma M., Constine L.S., Fajardo L.F., Kodama K., Mabuchi K., Marks L.B., Mettler F.A., Pierce L.J. (2010). Radiation-related heart disease: Current knowledge and future prospects. Int. J. Radiat. Oncol. Biol. Phys..

[68] Velensek V., Mazic U., Krzisnik C., Demšar D., Jazbec J., Jereb B. (2008). Cardiac damage after treatment of childhood cancer: A long-term follow-up. BMC Cancer.

[69] Erven K., Florian A., Slagmolen P., Sweldens C., Jurcut R., Wildiers H., Voigt J.-U., Weltens C. (2013). Subclinical cardiotoxicity detected by strain rate imaging up to 14 months after breast radiation therapy. Int. J. Radiat. Oncol. Biol. Phys..

[70] Hallahan D.E., Virudachalam S. (1997). Intercellular adhesion molecule 1 knockout abrogates radiation induced pulmonary inflammation. Proc. Natl. Acad. Sci. USA.

[71] Heidenreich P.A., Hancock S.L., Vagelos R.H., Lee B.K., Schnittger I. (2005). Diastolic dysfunction after mediastinal irradiation. Am. Heart J..

[72] Chang H.-M., Okwuosa T.M., Scarabelli T., Moudgil R., Yeh E.T.H. (2017). Cardiovascular Complications of Cancer Therapy. J. Am. Coll. Cardiol..

[73] Linhart A., Elliott P.M. (2007). The heart in Anderson-Fabry disease and other lysosomal storage disorders. Heart.

[74] Nagueh S.F. (2014). Anderson-Fabry Disease and Other Lysosomal Storage Disorders. Circulation.

[75] Takenaka T., Teraguchi H., Yoshida A., Taguchi S., Ninomiya K., Umekita Y., Yoshida H., Horinouchi M., Tabata K., Yonezawa S. (2008). Terminal stage cardiac findings in patients with cardiac Fabry disease: An electrocardiographic, echocardiographic, and autopsy study. J. Cardiol..

[76] Linhart A., Germain D.P., Olivotto I., Akhtar M.M., Anastasakis A., Hughes D., Namdar M., Pieroni M., Hagège A., Cecchi F. (2020). An expert consensus document on the management of cardiovascular manifestations of Fabry disease. Eur. J. Heart Fail..

[77] Ortiz A., Germain D.P., Desnick R.J., Politei J., Mauer M., Burlina A., Eng C., Hopkin R.J., Laney D., Linhart A. (2018). Fabry disease revisited: Management and treatment recommendations for adult patients. Mol. Genet. Metab..

[78] Ortiz A., Abiose A., Bichet D.G., Cabrera G., Charrow J., Germain D.P., Hopkin R.J., Jovanovic A., Linhart A., Maruti S.S. (2016). Time to treatment benefit for adult patients with Fabry disease receiving agalsidase β: Data from the Fabry Registry. J. Med. Genet..

[79] Séguéla P.-E., Iriart X., Acar P., Montaudon M., Roudaut R., Thambo J.-B. (2015). Eosinophilic cardiac disease: Molecular, clinical and imaging aspects. Arch. Cardiovasc. Dis..

[80] Zampieri M., Emmi G., Beltrami M., Fumagalli C., Urban M.L., Dei L.-L., Marchi A., Berteotti M., Tomberli A., Baldini K. (2021). Cardiac involvement in eosinophilic granulomatosis with polyangiitis (formerly Churg-Strauss syndrome): Prospective evaluation at a tertiary referral centre. Eur. J. Intern. Med..

[81] Slungaard A., Vercellotti G.M., Tran T., Gleich G.J., Key N.S. (1993). Eosinophil cationic granule proteins impair thrombomodulin function. A potential mechanism for thromboembolism in hypereosinophilic heart disease. J. Clin. Investig..

[82] Ogbogu P.U., Rosing D.R., Horne M.K. (2007). Cardiovascular Manifestations of Hypereosinophilic Syndromes. Immunol. Allergy Clin. N. Am..

[83] Gottdiener J.S., Maron B.J., Schooley R.T., Harley J.B., Roberts W.C., Fauci A.S. (1983). Two-dimensional echocardiographic assessment of the idiopathic hypereosinophilic syndrome. Anatomic basis of mitral regurgitation and peripheral embolization. Circulation.

[84] Anderson L., Pennell D. (2008). The role of endomyocardial biopsy in the management of cardiovascular disease: A scientific statement from the American Heart Association, the American College of Cardiology, and the European Society of Cardiology. Eur. Heart J..

[85] Limongelli G., Adorisio R., Baggio C., Bauce B., Biagini E., Castelletti S., Favilli S., Imazio M., Lioncino M., Merlo M. (2022). Diagnosis and Management of Rare Cardiomyopathies in Adult and Paediatric Patients. A Position Paper of the Italian Society of Cardiology (SIC) and Italian Society of Paediatric Cardiology (SICP). Int. J. Cardiol..

[86] Callegari A., Quandt D., Schmitz A., Klingel K., Balmer C., Dave H., Kretschmar O., Knirsch W. (2022). Findings and Outcome of Transcatheter Right Ventricular Endomyocardial Biopsy and Hemodynamic Assessment in Children with Suspected Myocarditis or Cardiomyopathy. Int. J. Environ. Res. Public Health.

[87] Mueller G.C., Michel-Behnke I., Knirsch W., Haas N.A., Abdul-Khaliq H., Gitter R., Dittrich S., Dähnert I., Uhlemann F., Schubert S. (2018). Feasibility, safety and diagnostic impact of endomyocardial biopsies for the diagnosis of myocardial disease in children and adolescents. EuroIntervention.

[88] Pophal S.G., Sigfusson G., Booth K.L., Bacanu S.-A., Webber S.A., Ettedgui J.A., Neches W.H., Park S.C. (1999). Complications of endomyocardial biopsy in children. J. Am. Coll. Cardiol..

[89] Brighenti M., Donti A., Giulia Gagliardi M., Maschietto N., Marini D., Lombardi M., Vairo U., Agnoletti G., Milanesi O., Pongiglione G. (2016). Endomyocardial biopsy safety and clinical yield in pediatric myocarditis: An Italian perspective: EMB in Pediatric Myocarditis. Cathet. Cardiovasc. Intervent..

[90] Jayaram N., Spertus J.A., Kennedy K.F., Vincent R., Martin G.R., Curtis J.P., Nykanen D., Moore P.M., Bergersen L. (2017). Modeling Major Adverse Outcomes of Pediatric and Adult Patients With Congenital Heart Disease Undergoing Cardiac Catheterization: Observations From the NCDR IMPACT Registry (National Cardiovascular Data Registry Improving Pediatric and Adult Congenital Treatment). Circulation.

[91] Mori H., Kogaki S., Ishida H., Yoshikawa T., Shindo T., Inuzuka R., Furutani Y., Ishido M., Nakanishi T. (2022). Outcomes of Restrictive Cardiomyopathy in Japanese Children―A Retrospective Cohort Study. Circ. J..

[92] Brunet-Garcia L., Roses-Noguer F., Betrián P., Balcells J., Gran F. (2021). Restrictive cardiomyopathy: Importance of early diagnosis. An. Pediatría.

[93] Wittekind S.G., Ryan T.D., Gao Z., Zafar F., Czosek R.J., Chin C.W., Jefferies J.L. (2019). Contemporary Outcomes of Pediatric Restrictive Cardiomyopathy: A Single-Center Experience. Pediatr. Cardiol..

[94] Rivenes S.M., Kearney D.L., Smith E.O., Towbin J.A., Denfield S.W. (2000). Sudden Death and Cardiovascular Collapse in Children With Restrictive Cardiomyopathy. Circulation.

[95] Newland D.M., Law Y.M., Albers E.L., Friedland-Little J.M., Ahmed H., Kemna M.S., Hong B.J. (2023). Early Clinical Experience with Dapagliflozin in Children with Heart Failure. Pediatr. Cardiol..

[96] Bogle C., Colan S.D., Miyamoto S.D., Choudhry S., Baez-Hernandez N., Brickler M.M., Feingold B., Lal A.K., Lee T.M., Canter C.E. (2023). Treatment Strategies for Cardiomyopathy in Children: A Scientific Statement From the American Heart Association. Circulation.

[97] Rath A., Weintraub R. (2021). Overview of Cardiomyopathies in Childhood. Front. Pediatr..

[98] Mehra M.R., Canter C.E., Hannan M.M., Semigran M.J., Uber P.A., Baran D.A., Danziger-Isakov L., Kirklin J.K., Kirk R., Kushwaha S.S. (2016). The 2016 International Society for Heart Lung Transplantation listing criteria for heart transplantation: A 10-year update. J. Heart Lung Transplant..

[99] Zangwill S.D., Naftel D., L’Ecuyer T., Rosenthal D., Robinson B., Kirklin J.K., Stendahl G., Dipchand A.I. (2009). Pediatric Heart Transplant Study Investigators Outcomes of children with restrictive cardiomyopathy listed for heart transplant: A multi-institutional study. J. Heart Lung Transpl..

[100] Fenton M.J., Chubb H., McMahon A.M., Rees P., Elliott M.J., Burch M. (2006). Heart and heart-lung transplantation for idiopathic restrictive cardiomyopathy in children. Heart.

[101] Bograd A.J., Mital S., Schwarzenberger J.C., Mosca R.S., Quaegebeur J.M., Addonizio L.J., Hsu D.T., Lamour J.M., Chen J.M. (2008). Twenty-year experience with heart transplantation for infants and children with restrictive cardiomyopathy: 1986–2006. Am. J. Transpl..

[102] Murtuza B., Fenton M., Burch M., Gupta A., Muthialu N., Elliott M.J., Hsia T.-Y., Tsang V.T., Kostolny M. (2013). Pediatric heart transplantation for congenital and restrictive cardiomyopathy. Ann. Thorac. Surg..

[103] Hetzer R., Javier M.F.D.M., Delmo Walter E.M. (2018). Role of paediatric assist device in bridge to transplant. Ann. Cardiothorac. Surg..

[104] Su J.A., Menteer J. (2017). Outcomes of Berlin Heart EXCOR^®^ pediatric ventricular assist device support in patients with restrictive and hypertrophic cardiomyopathy. Pediatr. Transpl..

[105] Maeda K., Nasirov T., Yarlagadda V., Hollander S.A., Navarathnum M., Rosenthal D.N., Chen S., Almond C.S., Kaufman B.D., Reinhartz O. (2019). Novel Trans-Septal Left Atrial VAD Cannulation Technique for Hypertrophic/Restrictive Cardiomyopathy. J. Heart Lung Transplant..

[106] Lorts A., Conway J., Schweiger M., Adachi I., Amdani S., Auerbach S.R., Barr C., Bleiweis M.S., Blume E.D., Burstein D.S. (2021). ISHLT consensus statement for the selection and management of pediatric and congenital heart disease patients on ventricular assist devices Endorsed by the American Heart Association. J. Heart Lung Transplant..

[107] Weller R.J., Weintraub R., Addonizio L.J., Chrisant M.R.K., Gersony W.M., Hsu D.T. (2002). Outcome of idiopathic restrictive cardiomyopathy in children. Am. J. Cardiol..

[108] Hoskote A., Carter C., Rees P., Elliott M., Burch M., Brown K. (2010). Acute right ventricular failure after pediatric cardiac transplant: Predictors and long-term outcome in current era of transplantation medicine. J. Thorac. Cardiovasc. Surg..

[109] Salzberg S.P., Lachat M.L., Von Harbou K., Zünd G., Turina M.I. (2005). Normalization of high pulmonary vascular resistance with LVAD support in heart transplantation candidates. Eur. J. Cardio-Thorac. Surg..

[110] Gulati G., Ruthazer R., Denofrio D., Vest A.R., Kent D., Kiernan M.S. (2021). Understanding Longitudinal Changes in Pulmonary Vascular Resistance After Left Ventricular Assist Device Implantation. J. Card. Fail..

[111] Schlein J., Riebandt J., Laufer G., Zimpfer D. (2021). Reversal of pulmonary hypertension in paediatric patients with restrictive cardiomyopathy. Interact. Cardiovasc. Thorac. Surg..

[112] Thangappan K., Morales D.L.S., Vu Q., Lehenbauer D., Villa C., Wittekind S., Hirsch R., Lorts A., Zafar F. (2021). Impact of mechanical circulatory support on pediatric heart transplant candidates with elevated pulmonary vascular resistance. Artif. Organs.

[113] Brodehl A., Gerull B. (2022). Genetic Insights into Primary Restrictive Cardiomyopathy. J. Clin. Med..

